# Best practices for guideline development in Critical Care

**DOI:** 10.62675/2965-2774.20250372

**Published:** 2025-02-21

**Authors:** Leticia Kawano-Dourado, Tyler Pitre, Dena Zeraatkar, Gordon Guyatt

**Affiliations:** 1 MAGIC Evidence Ecosystem Foundation Oslo Norway MAGIC Evidence Ecosystem Foundation - Oslo, Norway.; 2 HCor Research Institute Hcor-Hospital do Coração São Paulo SP Brazil HCor Research Institute, Hcor-Hospital do Coração - São Paulo (SP), Brazil.; 3 Universidade de São Paulo Instituto do Coração Faculdade de Medicina São Paulo SP Brazil Pulmonary Division, Instituto do Coração, Hospital das Clínicas, Faculdade de Medicina, Universidade de São Paulo - São Paulo (SP), Brazil.; 4 University of Toronto Division of Respirology Department of Medicine Toronto Canada Division of Respirology, Department of Medicine, University of Toronto - Toronto, Canada.; 5 McMaster University Department of Anesthesia Hamilton Ontario Canada Department of Anesthesia, McMaster University - Hamilton, Ontario, Canada.; 6 McMaster University Evidence and Impact Department of Health Research Methods Hamilton Ontario Canada Department of Health Research Methods, Evidence and Impact, McMaster University - Hamilton, Ontario, Canada.

## INTRODUCTION

Clinical practice guidelines (CPGs) facilitate evidence-based care.^(1)^ In critical care settings, where patients are often in life-threatening conditions and need fast and efficient care, trustworthy CPGs provide recommendations on the basis of the best current research evidence. For example, guidelines such as the Surviving Sepsis Campaign offer detailed recommendations on the timely administration of antibiotics and the use of vasopressors, which are crucial for managing sepsis.^(2)^ This enhances the quality and safety of patient care and reduces unwarranted variations in clinical practice.^(1-3)^

To achieve trustworthiness and transparency, guidelines must adhere to internationally accepted standards for guideline development.^(4)^ They must base recommendations on up-to-date and rigorous systematic reviews of the evidence, assess the certainty of evidence using comprehensive criteria that contain all the factors that may promote confidence in the evidence, consider patient values and preferences, use a structured and transparent approach to move from evidence to recommendations, and disclose and manage conflicts of interest.^(4,5)^ This article outlines best practices for guideline development in the context of Critical Care (Table 1).

**Table 1:** Best practices for guideline development

Core principles of guideline development
Systematic reviews – Clear eligibility criteria, comprehensive searches, and rigorous data extraction. – Assess certainty in evidence for each outcome using a systematic approach (e.g., GRADE).
Credibility of evidence – Use frameworks like GRADE to rate evidence certainty (high, moderate, low, very low) and systematically move from evidence to recommendations. – Recommendations should be based on a thorough appraisal of the evidence, avoiding selective or flawed interpretations.
Panel composition – Include patients with lived experience, clinicians, methodologists, and diverse representatives from the North and South Global geopolitical regions. – Address conflicts of interest with transparency and rigor.
Patient-centered values – Incorporate patient preferences, shared decision-making, and focus groups to ensure alignment with community priorities. – Address trade-offs in intervention outcomes.

GRADE - Grading of Recommendations Assessment, Development, and Evaluation.

### Core principles of guideline development

Rigorous systematic reviews specify clear eligibility criteria, conduct comprehensive searches, implement duplicate screening and data extraction, assess the risk of bias, and provide a quantitative synthesis of data via meta-analysis. Although the results from studies may seem impressive, their credibility can be undermined by bias, indirectness, inconsistency, imprecision, and publication bias. Therefore, systematic review authors should rate the certainty in evidence for each patient-important outcome on the basis of the entire body of evidence. Failing to base recommendations on systematic reviews and appraise the certainty of evidence can increase the risks of the selective use of evidence, disregarding important evidence, and ignoring important limitations of the selected evidence.

The use of corticosteroids in community-acquired pneumonia is an example wherein the systematic appraisal of the evidence coupled with the assessment of the quality of the evidence has helped to overcome controversy in the scientific literature. The controversy was largely due to inconsistencies in the results of trials dating back to 1956. Consequently, until recently, guidelines have continued to provide discordant recommendations.^(6,7)^ However, careful assessment of the evidence, including analysis of important heterogeneity and subgroup credibility (e.g., severe *versus* nonsevere), dose (e.g., higher *versus* lower), and molecule of administration (e.g., hydrocortisone *versus* methylprednisolone), has led to more confidence in recommendations in recent guidelines.^(8)^

**The GRADE (Grading of Recommendations Assessment, Development, and Evaluation) approach** is a robust and systematic way to appraise the certainty of evidence and the strength of recommendations in CPGs.^(9)^ The GRADE framework provides a systematic and transparent method for rating the certainty of evidence. In this framework, the certainty of evidence is categorized into four levels: high, moderate, low, and very low. This classification helps guideline developers understand the confidence they can have in the evidence when crafting recommendations.^(9,10)^

**Panel composition** should include not only traditional expert clinician-researchers but also – as our discussion of the need for optimal evidence synthesis has illustrated – collaboration with guideline methodologists. A demographically and culturally diverse guideline panel is another important element for guidelines of global interest. Participation by clinicians whose focus is day-to-day clinical practice, as well as patients with lived experience, provides crucial perspectives as panels move from evidence to recommendations. All panel members should ideally be free of both financial and nonfinancial conflicts of interest, and at the very least, guidelines must ensure that panelists fully disclose conflicts and implement strategies for their management.^(11)^

**Incorporating patients’ values and preferences** into guideline development is crucial to ensure that recommendations reflect the needs and priorities of the communities they serve. Indeed, all recommendations involve tradeoffs between the desirable and undesirable consequences of the alternative interventions under consideration, and value judgments about the importance of the outcomes—either explicit or implicit—underly every recommendation. To ensure patient values and preferences that inform recommendations requires consideration of research on patients’ values and preferences, reflection on shared decision-making that clinician panelists have undertaken in their practice, and ideally focus groups that bring patients together to reflect on their values.^(12)^

**Moving from evidence to recommendations**. Guideline developers can ensure that all relevant factors influencing recommendations are systematically considered by considering the GRADE evidence-to-recommendation frameworks. The frameworks facilitate the transparent and systematic assessment of all factors that may affect the recommendations, including the magnitude of benefits and harm, the certainty of the evidence, and patient values and preferences—the key issues for every recommendation—and often, in addition, resource implications, equity, acceptability, and feasibility. Transparent documentation of the judgments made within this framework can prevent guideline users from being unclear about the underlying rationale for the recommendations.

### Challenges

The implementation of best practices in CPG development requires methodological expertise, human and financial resources, and requirements that might become hurdles in resource-constrained settings, leading to increased global health inequities.^(13)^ A potential solution in resource-constrained settings would be to adapt international CPGs, a process that still requires methodological expertise but is less human- and resource-intensive.^(14)^

An additional challenge is that trustworthy CPGs are useless if they are not implemented in clinical practice. Resistance to change is a significant barrier to the implementation of new evidence-based practices in every area of medicine and does not differ across critical care settings. This resistance often stems from a combination of individual, interpersonal, and organizational factors, such as fear, uncertainty, lack of trust, and inadequate communication.^(15)^ Strategies for overcoming barriers to adoption in the critical care setting include encouraging collaboration among different health care professionals so that the guidelines can be adapted to the practices of all stakeholders.^(15)^ Additionally, tailoring guidelines to the local context may reduce resistance to guideline adoption.^(16)^

The complexity of guideline documents represents another potential barrier to adherence. Simplifying documents and providing clear, concise, and actionable recommendations will increase accessibility and usefulness. Visual aids, flowcharts, and summaries can be particularly useful in fast-paced environments, such as ICUs.^(17)^

## Future directions and conclusions

Despite these challenges, methods for guideline development have advanced in the past two decades. A notable recent innovation is the adoption of living clinical practice guidelines.^(18)^ During the COVID-19 pandemic, evidence users, including clinicians, guideline developers, and policy-makers, had difficulty distinguishing trustworthy from untrustworthy evidence and interpreting the results of studies within a landscape of evidence that was changing with unprecedented rapidity. To address these challenges, organizations produced living guidelines that were updated as new evidence emerged.^(19)^ Since then, organizations have applied living guideline methods to other disease areas.^(20)^ This approach overcomes the limitations of conventional guidelines by allowing more rapid revision of recommendations on the basis of emerging evidence.

Other upcoming innovations in guideline development include the incorporation of utilities for the consideration of patient values and preferences, more efficient and comprehensive consideration of panel conflicts of interest, and the integration of advanced technology and artificial intelligence to facilitate more efficient evidence synthesis.^(21-23)^
